# Housing land transaction data and structural econometric estimation of preference parameters for urban economic simulation models

**DOI:** 10.1016/j.dib.2015.09.047

**Published:** 2015-10-13

**Authors:** Geoffrey Caruso, Jean Cavailhès, Dominique Peeters, Isabelle Thomas, Pierre Frankhauser, Gilles Vuidel

**Affiliations:** a Institute of Geography and Spatial Planning, University of Luxembourg, Luxembourg; b UMR 1041 CESAER-INRA Institut National de Recherche Agronomique, Dijon, France; c CORE Center for Operations Research and Econometrics, Université catholique de Louvain, Louvain-la-Neuve, Belgium; d FRS-FNRS, Fonds National de la Recherche Scientifique, Université catholique de Louvain, Louvain-la-Neuve, Belgium; e UMR ThéMA, CNRS, Centre National de la Recherche Scientifique, University of Franche-Comté, Besançon, France

## Abstract

This paper describes a dataset of 6284 land transactions prices and plot surfaces in 3 medium-sized cities in France (Besançon, Dijon and Brest). The dataset includes road accessibility as obtained from a minimization algorithm, and the amount of green space available to households in the neighborhood of the transactions, as evaluated from a land cover dataset. Further to the data presentation, the paper describes how these variables can be used to estimate the non-observable parameters of a residential choice function explicitly derived from a microeconomic model. The estimates are used by Caruso et al. (2015) to run a calibrated microeconomic urban growth simulation model where households are assumed to trade-off accessibility and local green space amenities.

**Specifications Table**

**Table:** 

Subject area	Economics, Geography
More specific subject area	Urban economics/geography, Econometrics
Type of data	Tables
How data was acquired	Official housing land transaction data; commuting distance computation with Odomatrix from the precise location of the transaction and location of the job center [6]; Aggregation of CORINE land cover data [4] to municipal level for neighbourhood density
Data format	Filtered and analyzed
Experimental factors	Land transactions located within specific urban areas (‘aire urbaine’) and selected after an upper limit of plot surfaces and atypical records removal
Experimental features	Data selection, parametric specification for observable parameters, and econometrics to estimate non-observable preferences for residential consumption and local green amenities
Data source location	France. Urban area of Dijon, Besançon and Brest
Data accessibility	Data with this article.

**Value of the data**

• This data is a cleaned set of housing land transactionson medium-sized French urban areas for benchmarking urban analysis and models with local and distance effects.
• An econometricmethod is described to show how one can estimate the non-observable parameters of a residential choice function explicitly derived from a microeconomic model.
• The resulting estimates can also be used to calibrate other urban simulation models directly where central accessibility and local density (green amenities) are traded-off by households.

## 1 Data, experimental design, materials and methods

### 1.1 Data

*We share a dataset* of housing land transaction for medium-sized French urban areas. The data contains the main characteristics of plots, i.e. price and surface, plus variables computed from their precise geographic location (distance and neighborhood density) while preserving anonymity of the people involved in the transaction. This data has been acquired and selected as described below in order to allow for parameters calibration of an urban growth simulation model (using structural equations of the economic model, see Section 1.2)

#### 1.1.1 Acquisition and selection

Housing represents the largest share of household’s expenditure and vary between a quarter and half of disposable income in most Western countries (around 30% in France). The housing sector is also at the origin of important economic booms (around 2000) or severe crises (in 2008 for example). Despite the importance of this sector, econometric research is still rare about housing, in particular because of a lack of good quality statistical data on realized housing transactions.

France is one of those countries where data exist and are made available to researchers. Cadasters exist since the early 19th Century and all private plots of land are recorded, mapped and georeferenced precisely. The central fiscal administration records all rights and transactions applying to plots and properties. Each transaction is recorded by a notary, i.e. a public agent specialized in real estate transactions and whose mission includes the transmission of data to the fiscal administration. Notaries are required by law to send information to a database named PERVAL. This permits the publication of a dataset with a high coverage rate (the managers announce a 75% coverage of all land and housing change and transactions) and an increasing quality of quantitative and qualitative information. Notaries send the following information to PERVAL:

1. ID and date of transaction.
2. Characteristics of the property: nature (land, house,...), postal address, cadastral references, surface of plot, complementary information for houses and apartments (surface, building date, equipment, etc.).
3. Prices: net prices and related transaction costs.
4. Characteristics of the seller and of the buyer: nature (physical person, firm,…), profession, gender, family status, age, place of residence, nationality.

For the needs of the research by Caruso et al. [2] (G. Caruso, J. Cavailhès, D. Peeters, I. Thomas, P. Frankhauser, G. Vuidel, Greener and larger neighborhoods make cities more sustainable! A 2D urban economics perspective, Computers Environment and Urban Systems, 2015, 54, pp. 82–94.) housing land transaction data were extracted from PERVAL for 3 medium-size urban areas in France: Besançon, Brest and Dijon. According to the 2010 zoning by the Institut National de la Statistique et des Etudes Economiques (INSEE), a large or medium-sized urban area is made of a compact core, offering at least 5000 jobs, and a periurban belt of towns and villages scattered in the countryside where at least 40% of the working population commutes daily to the core.

The data relates to developable land where no building is constructed yet at the time of the transaction, so that land effects can be strictly separated from any building characteristics effects. The dataset includes transactions from the years 2000, 2002, 2004 and 2006. Records with missing attributes we not considered. A typical transaction records were removed from an analysis of the distribution of prices and surfaces. The dataset was then further filtered to keep only over-the-counter transactions (‘gré à gré’) thus avoiding large real estate development projects and potential artifacts on the value of individual plots.

Plots surface and transaction date and prices were extracted from PERVAL. Accessibility and neighborhood attributes were then added and computed from the geographical location of the plots.

In terms of accessibility, the distance between each plot and the center (CBD) of each urban area is considered. A minimum path computation has been performed with the Odomatrix software [6] using the road network dataset ‘Route500^©^’ from the French Geographic Institute (IGN). The algorithm accounts for geographical context (altitudes, slopes, urban agglomeration or countryside environment, etc.) and traffic conditions (off-peak or peak hours). The tool chooses the itinerary that minimizes total travel time. Travel time is expressed in minutes of driving a car along the road network to the closest urban center. The computation is undertaken at the scale of municipalities (smallest administrative unit), which implies that if a plot falls within the extent of the municipality where the center is located, the distance is set to 0.

The neighborhood effects considered in Caruso et al [2] (G. Caruso, J. Cavailhès, D. Peeters, I. Thomas, P. Frankhauser, G. Vuidel, Greener and larger neighborhoods make cities more sustainable! A 2D urban economics perspective, Computers Environment and Urban Systems, 2015, 54, pp. 82–94.) depend on the density of built-up land within a given neighborhood window around each plot. Given the theoretical assumptions of the model, the size of the window must account for both the view of green space and for social contacts. According to the literature (e.g. [1], [7], or [5], In Press), only the first few hundred meters around a residence matter for green amenities. In terms of social contacts, the interactions considered as local externalities must be costless and therefore should correspond to a walkable catchment area (`ped-shed׳). The density is obtained from the CORINE Land Cover 2006 data [4], which, despite potential underestimates, provides a reasonable value for the share of land devoted to urban or transportation by municipality. The neighborhood where households enjoy amenities is assumed to correspond to the extent of a municipality. This is quite a realistic assumption since the average surface of a municipality is 1179 ha, which is equivalent to a square with a side of 1.1 km or to a circle with a radius of 600 m. It is reasonable to assume that inhabitants benefit from green amenities within that neighborhood and can walk across for social contacts. The median number of inhabitants of a municipality in our study area is 1600, which corresponds to 700 households. This is a reasonable assumption for a social interaction potential.

#### 1.1.2 File description

This *Data in brief* includes Supplementary material in the file ‘Caruso_etal_DIB_data’ (csv format) for the following variables and the 6284 transaction records selected as described above in this article

transaction records selected as described previously:

1. *URBAN*: the name of the urban area (Dijon, Besançon, Brest).
2. *YEAR*: the year the transaction was recorded (2000, 2002, 2004 or 2006).
3. *SURFACE*: the surface of plots, in square meters.
4. *RENT*: the annualized rent obtained from the price of a transaction (using a 4% interest rate), in Euro as of 2006 (after application of price index).
5. *DISTANCE*: the distance in minutes to Dijon, Besançon or Brest center (CBD), resulting from Odomatrix software.
6. *DENSITY*: the share of land devoted to urban or transportation uses within the municipality where the plot has been sold.

### 2 Urban model calibration

The model proposed by Caruso et al. [2] (G. Caruso, J. Cavailhès, D. Peeters, I. Thomas, P. Frankhauser, G. Vuidel, Greener and larger neighborhoods make cities more sustainable! A 2D urban economics perspective, Computers Environment and Urban Systems*,* 2015, 54, pp. 82–94.) builds on the maximization of a utility function by households subject to a budget constraint. The growth of the city results from applying the indirect utility function to find out what is the best location for a household at each iteration of the model. The parameters of the indirect utility function need numerical value. While some of them are observable and can be simply set from statistics, preference parameters are not directly observable. We show below how the behavioral equations of the model can be used to infer preference parameters econometrically, prior to any simulation.1 In fact, the maximization program holds explicitly plot size and rent levels, which both can be observed. Non-observable preference parameters can therefore be estimated directly from the data described above and those structural equations.

We first describe these equations as they derive from the decision program of households in Caruso et al. (In Press) and the value of observable parameters. Second, we present the estimation of the non-observable preference parameters. The resulting set of parameters is used to run the benchmark simulation in Caruso et al. (In Press)

### 2.1 Equations and observed parameters

The following rent function results from the maximization of the microeconomic program of households proposed in Caruso et al. (In Press, see Eq. (2), after dropping subscripts for locations for clarity):

(1) $${R=(Y-\theta D)}^{1/\alpha }U^{-1/\alpha }e^{-\beta \rho /\alpha }$$

where *R* is the rent per surface unit of land; *Y* the annual household income; *D* the distance to the center (CBD) of the urban area; θ the cost of commuting per unit of distance*; U* the reference utility level; ρ the neighborhood density; and α and β are preference parameters to be estimated.

In addition to equilibrium rent, one can also obtain from the maximization solution the following two equations, respectively the share of residential land consumption in the available budget of households

(2) RS=α(Y−θD)

and the surface of residential plots:

(3) $$S={(Y-\theta D)}^{1-1/\alpha }U^{1/\alpha }e^{\beta \rho /\alpha }$$

Within those equations, four variables are obtained from the transactions database described above and have specific value for each transaction: land rent, *R* (see RENT in database); land consumption, *S* (see SURFACE); the distance to the CBD, D (see DISTANCE); and the local density, ρ (see DENSITY).

The annual household income, *Y*, is assumed to be homogenous across households and approximated to 29,000 € per year based on data from the Ministry for Budget and from INSEE for those specific urban areas. Tests have been performed and show stability of the results to changing the defined level of income.

The generalized unit transport cost, θ, is made of a direct monetary cost (from the fiscal administration) of 0.40 € per km, and of an opportunity cost of time, know from experts׳ assessment to be 0.15 € per minute. We obtain a generalized annual cost of 330 € per minutes, after assuming 200 annual return journeys to work for 1.5 worker per household.

Among the other parameters, *U* is a constant utility level and does not relate to a particular behavior. It will be captured within the intercept estimates. The other parameters, α, β are non-observable parameters to be estimated econometrically. α is attached to the consumption of housing land, i.e. RS in Eq. (2) or RENT*SURFACE in the data. β is attached to the preference for a greener neighborhood (with respect to social interactions), which itself is supposed to be inversely decreasing with local density, ρ in Eq. (3) or DENSITY in the data. Given the functional form chosen (see direct utility in Caruso et al., In Press), a positive α and a negative β are expected.

#### 2.2 Econometric estimates

The estimation is done in two stages: first, α is obtained from the share of residential land consumption in the available budget equation above (Eq. (2)) and denoted as $\hat{\alpha }$. Then Eqs. (1), (3) are transformed in such a way that $\hat{\alpha }$ moves to the left-hand side of the equation and the coefficient β for density can be estimated:

(4) $$\hat{\alpha }\ln\;R-\ln\left(Y-\theta D\right)=-\ln\;U-\beta \rho$$

(5) $$\hat{\alpha }\ln\;S-\hat{\alpha }\ln\;\hat{\alpha }-(\hat{\alpha }-1)\ln\left(Y-\theta D\right)=\ln\;U+\beta \rho$$

Control dummies for the transaction year and urban area are included in the estimation (with 2006 and Dijon as references). The estimation is made without intercept. Results are displayed in Table 1.

We obtain $\hat{\alpha }=$0.06925, which is quite low for a parameter that represents the share of residential expenses in the consumer׳s budget, but this is due to the fact that we only consider raw land rent: the price of the building is integrated within the general composite good consumed by households since it is footloose, i.e. the price of materials and labor for building detached houses typically does not depend on location.

It is then possible to estimate Eqs. (4), (5). Results are displayed in Table 2, Table 3 below. The parameters estimated from those structural equations are provided in the tables below. Overall fits are rather low (*R*^2^=0.28 and 0.34), which shows that such a simple specification cannot cover all complex aspects of the land market reality. Nevertheless, β estimates are very significant and very close values are obtained from the two equations: −0.38 and −0.42. A value of β=−0.40 is chosen for the benchmark simulation in Caruso et al. [2].

## Figures and Tables

**Table 1:** Share of residential land consumption. Coefficient estimates. Dependent variable: RENT*SURFACE (see Eq. (2)).

**Variable**	**Coefficient**	**Student*****t***
Y−θ*DISTANCE	**0.06925**	110.68
*n=*6284		
*Adj. R sq.=*0.66		

**Table 2:** Local density preference. Coefficient estimates from rent equation. Dependent variable: $\hat{\alpha }*\ln\mathrm{RENT}-\ln\left(Y-\theta *\mathrm{DISTANCE}\right)$ (see Eq. (4)).

**Variable**	**Coefficient**	**Student*****t***
Intercept	−9.83195	−2415.70
DENSITY	**−0.37721**	−37.77
BESANCON	−0.08496	−20.00
BREST	0.02182	5.17
DIJON	reference	
YEAR 2000	−0.07061	−15.62
YEAR 2002	−0.05660	−12.10
YEAR 2004	−0.03642	−8.03
YEAR 2006	reference	
*n=*6284		
*Adj. R sq.*=0.28		

**Table 3:** Local density preference. Coefficient estimates from land consumption equation. Dependent variable: $\hat{\alpha }*\ln\mathrm{SURFACE}-\hat{\alpha }*\ln\hat{\alpha }-(\hat{\alpha }-1)*\ln\left(Y-\theta *\mathrm{DISTANCE}\right)$ (see Eq. (5)).

**Variable**	**Coefficient**	**Student*****t***
Intercept	−9.82388	−2695.82
DENSITY	**−0.42474**	−47.51
BESANCON	−0.08146	−21.42
BREST	0.02947	7.80
DIJON	reference	
YEAR 2000	−0.03258	−8.05
YEAR 2002	−0.02122	−5.07
YEAR 2004	−0.01645	−4.05
YEAR 2006	reference	
*n*=6284		
*Adj. R sq.*=0.34		

## Footnotes

1 Another approach was taken by Caruso and Hilal [3] who use the land market outcome of simulations and analyze how well it fits observed rents and land consumptions. This calibration methods requests to loop back and forth between simulation results and parameters, conversely to the econometric estimation undertaken here.
